# Evaluation of Germplasm Resources and Microbial Diversity Among Different Geographical Provenances of *Tamarindus indica* L.

**DOI:** 10.3390/microorganisms14050983

**Published:** 2026-04-28

**Authors:** Shuangyuan Yu, Wenjie Tang, Zibo Song, Yuehan Wang, Weijie Gao, Yi Su, Xiaoming Yang, Yihe Zhao, Tingting Dai

**Affiliations:** 1 College of Forestry and Grassland & College of Soil and Water Conservation, Nanjing Forestry University, Nanjing 210037, China; ysy199949@163.com (S.Y.); m15951872958@163.com (W.T.); wangyuehan@njfu.edu.cn (Y.W.); gaotangbuer@njfu.edu.cn (W.G.); xmyang@njfu.edu.cn (X.Y.); 2 Yunnan Provincial Key Laboratory of Applied Technology for Special Forest Fruits, Yuxi 653100, China; szb1031@163.com; 3 Yunnan Academy of Forestry and Grassland, Kunming 650204, China; suyi@yafg.ac.cn

**Keywords:** tamarind, microorganism, host–microbial interactions, geographical provenances

## Abstract

*Tamarindus indica* L. is a species of tree with high economic value. However, research on its associated bacterial communities is limited, and no microbial fertilizer has yet been developed specifically for tamarind. In this study, we selected 20 geographical provenances of tamarind as experimental materials, evaluated their germplasm resources, and investigated the correlation between plant traits and associated bacterial communities under grafting conditions. Provenances YM2 and BS21 produced the largest fruits, while all physiological indices showed significant variability among the tested accessions. Microbial samples from the phyllosphere and rhizosphere were collected from these 20 provenances, and 16S rRNA gene sequencing was conducted to compare microbial communities. The differences in rhizosphere microbiota among different samples were more significant than those in phyllosphere microbiota; subsequently, an in-depth investigation was conducted on the relationships between rhizosphere bacterial communities and various traits under these grafting conditions. Through correlation analysis, significant correlations were identified between some microbial phyla and the traits of tamarind under these grafting conditions. Under the current grafting conditions, variations in the rhizosphere microbiome were associated with tamarind provenances. However, due to the constraints of the experimental design, the potential influences of rootstock genotypes and scion–rootstock signal transduction could not be excluded. Nevertheless, through the unification of rootstock sources and the design of correlation analysis, this study has initially verified the dominant association between scion provenances and microbial communities.

## 1. Introduction

The leaves and roots of plants are colonized by diverse bacterial communities that influence their fitness and evolutionary trajectories. Numerous studies have demonstrated that the intricate interactions between plant species and their associated microbiota can modulate developmental outcomes [1,2,3,4]. In recent years, the essential role of microorganisms in plant health has been increasingly recognized (Table 1). In 2024, Ahsan T. developed a microbial inoculant that has been shown to enhance peanut growth and improve yield, prompting a surge in research on microbial inoculants. While it was reported as early as 1990 that arbuscular mycorrhizal fungi (AMF) could enhance tamarind’s growth, there have been no documented cases of specific bacteria with targeted growth-promoting effects on tamarind. Effective microbial inoculants for tamarind have also yet to be developed.

Tamarind (*Tamarindus indica* Linn.) is native to West Africa and was spread by Arab merchants due to the pleasant sour taste of its fruit. It is now distributed throughout tropical and subtropical regions [11]. Due to the unique flavor of the fruit, the commercial processing of its pulp into beverages has become a key industry in production areas, with related juice extraction techniques under continuous study [12]. Tamarind is known for the distinctive flavor of its pulp and also possesses geographical distinct traits with potential applications and value. Tamarind seeds are rich in antioxidant-active compounds. The polysaccharides extracted from these seeds exhibit exceptional pH stability (pH 1–10) in aqueous solutions, demonstrating their potential use as thickeners and in tobacco products [13,14]. Tamarind polysaccharides may also serve as delivery agents in the treatment of diseases such as diabetes [15,16]. India alone produces nearly 300,000 tons of tamarind pods annually, while the Philippines exports tamarind worth nearly 100,000 USD each year [17]. Recent studies indicate that tamarind leaves contain flavonoids and other antioxidant-active compounds with anti-inflammatory and antioxidant properties [18,19]. The compounds present in tamarind are also considered safe for human consumption [20,21]. In Eastern Uganda, researchers surveyed participants on 18 uses of tamarind and found that indigenous knowledge aligns with scientifically documented nutritional and medicinal properties of the plant [22]. Traditional Chinese medical research documents diverse applications of tamarind, suggesting an underexplored potential that warrants further investigation. In Benin, tamarind contributes significantly to household income, accounting for between 8.8% and 56.4% of average household earnings [23]. In conclusion, tamarind is a tree species of high economic value; so, microbial screening aimed at enhancing its yield is highly significant. The objectives of this study are as follows: (a) evaluate the germplasm resources of tamarind from different geographical provenances grown in a common garden; (b) analyze the composition and diversity characteristics of rhizosphere and phyllosphere microbial communities associated with tamarind from different provenances; (c) explore whether there is any correlation between the microbial community characteristics of tamarind and plant phenotypic, physiological traits. To address this issue, we conducted a field-based common-garden experiment. Maoduoli Manor is located in Xinping County, Yuxi City, Yunnan Province. We hypothesized that the composition and diversity of rhizosphere and phyllosphere microorganisms would differ significantly among different provenances, and that these differences would be closely related to plant growth and physiological traits.

## 2. Materials and Methods

**Figure 1:**
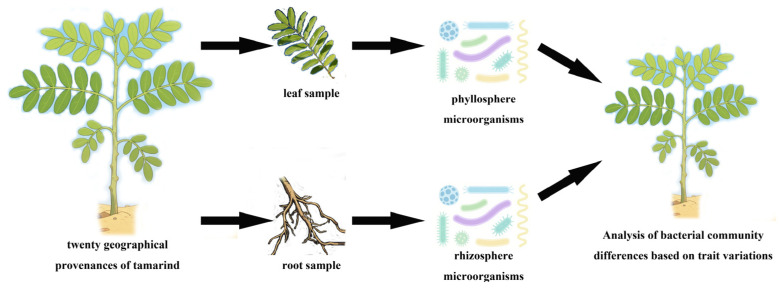
Experimental procedure flowchart.

The geographical coordinates of Maoduoli Manor are 23.76° N, 101.79° E, and it is located at an altitude of 720 m. The minimum annual temperature is 6 °C, with a maximum reaching 44 °C, with an average relative humidity of 63.3%. The manor hosts over 200 geographical provenances of tamarind, which were collected from plantations worldwide for research purposes. In 2013, various geographical provenances were grafted onto two-year-old Tamarindus indica seedlings of the same origin as rootstocks in the germplasm resource nursery. We selected 20 geographical provenances of tamarind (Table 2), with three clones per provenance. We collected rhizosphere soil and fresh living leaves in sterile centrifuge tubes, and the fruits were collected using nylon mesh bags. We then extracted rhizosphere and phyllosphere bacterial communities from the collected roots and leaves. Next, we used 16S ribosomal RNA gene sequencing to amplify microbial DNA and create a comprehensive microbial database. This database was subsequently used to investigate the complex relationships between plant traits and both rhizosphere- and leaf-associated microbial communities (Figure 1).

### 2.1. Molecular Experimental Procedure for Bacterial Communities in Plant-Related Samples

#### 2.1.1. Rhizosphere Soil and Phyllosphere Suspension Precipitate Collection

Following Yannai’s definition of plant rhizosphere soil [24], the collected roots were rinsed with PBS buffer at 4 °C. Subsequently, samples were centrifuged at 4000 RPM for 30 min at 4 °C to remove the supernatant and obtain the soil sediment. A total of 10 g of leaves was collected and immersed in sterile PBS buffer (pH 7.4) with a volume sufficient to fully submerge the leaves. The mixture was incubated on a shaker at 200 rpm for 1 h. After removing the leaves, the resulting PBS buffer was filtered through two layers of sterile gauze to remove debris. Subsequently, the filtrate was centrifuged at 5000× *g* for 10 min at 25 °C. The supernatant was discarded, and the phyllosphere precipitate was collected.

#### 2.1.2. Library Loading and Sequencing

For the preprocessed samples, nucleic acids were extracted using the MagBeads FastDNA Kit for Soil (116564384) (MP Biomedicals, Irvine, CA, USA). The extracted DNA was analyzed via 0.8% agarose gel electrophoresis to assess molecular size and was quantified using a Nanodrop spectrophotometer (Thermo Fisher Scientific, Wilmington, DE, USA) . PCR amplification was performed using specific primers targeting the V3–V4 hypervariable regions of the bacterial 16S rRNA gene: 338F (5′-barcode+ACTCCTACGGGAGGCAGCA-3′) and 806R (5′-GGACTACHVGGGTWTCTAAT-3′). The barcode in the forward primer is a 7–10 base oligonucleotide sequence used to differentiate samples within the same library (The PCR reaction system is shown in Table A1. PCR products were purified using VAHTS™ DNA Clean Beads (0.8× volume)(Vazyme Biotech Co., Ltd., Nanjing, China), washed with 80% ethanol, and eluted in 25 μL buffer. Purified amplicons were quantified using PicoGreen (Thermo Fisher Scientific, Waltham, MA, USA) on a BioTek FLx800 (BioTek Instruments, Inc., Winooski, VT, USA), normalized, and pooled. Libraries were prepared using the Illumina TruSeq Nano Kit (Illumina, Inc., San Diego, CA, USA), involving end repair, A-tailing, adapter ligation, and purification with AMPure XP Beads (Beckman Coulter Life Sciences, Indianapolis, IN, USA). Final size selection was conducted via 2% agarose gel electrophoresis. Libraries were validated in an Agilent Bioanalyzer (Agilent Technologies, Inc., Santa Clara, CA, USA), quantified using QuantiFluor (Promega Corporation, Madison, WI, USA), pooled at ≥2 nM, denatured with NaOH, and sequenced in MiSeq (600 cycles) platforms (Illumina, Inc., San Diego, CA, USA), targeting 200–450 bp fragments for optimal coverage [25].

#### 2.1.3. Quality Control and Data Processing

DADA2 Sequence Denoising: Sequence quality control and denoising were performed using QIIME2 version 2024.5, with the following detailed steps: (1) Sample-specific barcode sequences (7–10 bp) at the 5′-end of forward reads were trimmed using the qiime cutadapt trim-paired command. Primer trimming: The qiime cutadapt trim-paired command was invoked to trim primer sequences, with the forward primer (5′-ACTCCTACGGGAGGCAGCA-3′) and reverse primer (5′-GGACTACHVGGGTWTCTAAT-3′) specified. The primer match length threshold (--p-overlap = 10) and error rate (--p-error-rate = 0.1) were set, and sequences with unmatched primers or insufficient primer match length were discarded. (2) DADA2 denoising: The qiime dada2 denoise-paired command was executed for sequence quality control, denoising, paired-end merging, and chimera removal. Truncation lengths (trunc-len-f = 280, trunc-len-r = 220) and maximum expected error values (max-ee-f = 2, max-ee-r = 3) were configured, for rhizosphere microbiota, the “--p-chimera-method = consensus” parameter was used for chimera removal. Using the same consensus parameter for phyllosphere microbiota resulted in inadequate data filtering; therefore, the “--p-chimera-method = pooled” parameter was adopted for phyllosphere microbiota; all other parameters were set to default. (3) The above steps were performed separately for each sequencing library to ensure analytical consistency across libraries. (4) After denoising, the qiime feature-table merge command was used to merge Amplicon sequence variants (ASVs) representative sequences and ASV abundance tables across all libraries. The qiime feature-table filter-features command was then applied to remove singleton ASVs (ASVs with a total sequence count of 1 across all samples). (5) Quality control criteria: Only sequences with Q30 ≥ 90%, a length of 250–350 bp, and no ambiguous bases (N) were retained. Samples with fewer than 1000 filtered valid sequences were excluded from downstream analyses. Reads before and after denoising are provided in Table A2 and Table A3. The present study employed DADA2 denoising to obtain amplicon sequence variants (ASVs), and operational taxonomic unit (OTU) clustering analysis was not performed [26,27]. Using the SILVA 138.1 Ref NR 99 database, taxonomic annotation of amplicon sequence variant (ASV) representative sequences was performed via the qiime feature-classifier classify-sklearn tool in QIIME2. A sequence similarity threshold of 97% and a taxonomic confidence threshold of 0.7 were set, yielding taxonomic information for each ASV at the phylum, class, order, family, and genus levels.

For beta diversity analysis, the Bray–Curtis distance matrix was calculated using the vegdist function from the vegan package -(version 2.6.6) in R (version 4.3.3). UPGMA clustering was conducted with the hclust function from the stats package, and the results were visualized using the ggtree package. Simultaneously, PERMANOVA analysis was performed using the adonis function from the vegan package (number of permutations = 999), with effect sizes (R^2^) and *p*-values reported. Principal Coordinate Analysis (PCoA) plots were generated using the ggplot2 package, with sample groups and variance explained by each axis labeled.

For alpha diversity analysis, the maximum rarefaction depth was set to 95% of the sequence count of the sample with the lowest sequencing depth. Ten depth values were evenly selected between this maximum depth and the minimum sequencing depth. At each depth, sequences were randomly rarefied 10 times using the vegan package (version 2.6.6) to calculate alpha diversity indices, including Chao1, Shannon, Simpson, and Observed_species. Rarefaction curves were plotted accordingly. All visualizations were performed in R (version 4.3.3).

### 2.2. Assay of Superoxide Dismutase (SOD) and Peroxidase (POD)

SOD can eliminate superoxide anion radicals produced by riboflavin upon illumination [28]. Simultaneously, the POD-catalyzed oxidation of specific substrates by hydrogen peroxide provides the basis for determining the SOD and POD activities [29]. The reagent kit used for SOD and POD assays was supplied by Komson Biotechnology Co., Ltd. (Nanjing, China).

### 2.3. Determination of Flavonoid

**Figure A1:**
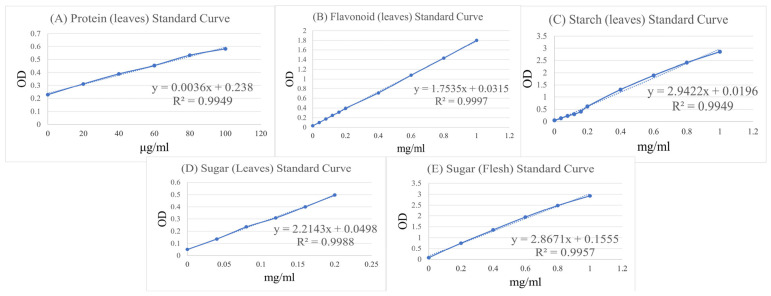
The standard curves used in the experiment. (**A**) Standard curves for soluble protein content in leaves (0~100 μg/mL). (**B**) The standard curves for flavonoid content in leaves (0~1 mg/mL). (**C**) The standard curves for starch content in leaves (0~1 mg/mL). (**D**) The standard curves for sugar content in leaves (0~0.2 mg/mL). (**E**) The standard curves for sugar content in flesh (0~0.2 mg/mL).

Fresh leaves (0.1 g) were carefully weighed and finely homogenized in 4 mL of 75% ethanol using a pre-cooled mortar and pestle. The homogenate was transferred to a centrifuge tube and centrifuged at 10,000 RPM for 10 min at 25 °C; the resulting supernatant was collected and retained. The remaining precipitate was resuspended in 4 mL of fresh 75% ethanol, subjected to ultrasonication for 15 min to improve extraction efficiency, and then centrifuged again at 10,000 RPM for 10 min at 25 °C to obtain a second supernatant. Both supernatants were pooled, transferred into a volumetric flask, and treated with 0.1 g of activated carbon (Fujian Yuanli Activated Carbon Co., Ltd., Nanping, China) via vigorous shaking before filtration. The resulting filtrate was adjusted to a final volume of 10 mL with 75% ethanol to obtain the test solution [30]. For quantification, 1 mL of the test solution was pipetted into a test tube, followed by the sequential addition of 0.3 mL of 5% sodium nitrite, 0.3 mL of 10% aluminum nitrate, and 2 mL of 4% sodium hydroxide [31]. After thorough mixing, the reaction mixture was allowed to settle at 25 °C to ensure complete reaction. The absorbance of the final solution was measured at 510 nm using a UV–visible spectrophotometer, with a reagent blank serving as the reference. The concentration of the analyte was determined by comparing the absorbance value with that obtained from a standard calibration curve (Figure A1).

### 2.4. Determination of Soluble Protein

To determine soluble protein content, 0.1 g of fresh leaves was precisely weighed and placed in a pre-cooled mortar. An aliquot of 4 mL ultrapure water was added, and the sample was thoroughly ground to obtain a homogenate. The homogenate was then centrifuged at 10,000 RPM for 10 min at 4 °C, the supernatant was made up to a final volume of 5 mL. For quantification, 1 mL of the test solution was pipetted into a test tube, followed by the sequential addition of 5 mL of Coomassie Brilliant Blue solution (Sangon Biotech Co., Ltd., Shanghai, China). Absorbance was measured at 595 nm using a spectrophotometer. Finally, the absorbance value was compared with a pre-established BSA standard curve (Figure A1) to quantify the soluble protein content [32].

### 2.5. Determination of Sugar and Starch

Sugar and starch contents were measured using the method described by Akiko Ito with moderate modifications [33]. Briefly, 0.1 g of fresh leaf tissue was homogenized in 4 mL of 75% ethanol, and the homogenate was centrifuged at 10,000 RPM for 10 min at 25 °C to extract the supernatant. The remaining residue was re-extracted with an additional 4 mL of 75% ethanol via sonication at 60 °C for 15 min, followed by centrifugation at 10,000 RPM for 10 min at 25 °C. The two resulting supernatants were pooled, decolorized with 0.1 g of activated carbon through vigorous shaking, filtered, and adjusted to a final volume of 10 mL to obtain the sugar extract. For starch extraction, the residue from the sugar extraction step was mixed with 3 mL of boiling water and heated for 30 min to induce gelatinization. After cooling, 2 mL of 9.2 mol/L perchloric acid was added, and the mixture was shaken for 15 min before centrifugation. This extraction step was repeated, and the resulting supernatants were combined and brought to a final volume of 10 mL to prepare the starch extract. For quantification, 1 mL of each extract (sugar or starch) was mixed with 5 mL of 0.1% anthrone reagent in 80% sulfuric acid, heated in a boiling water bath for 30 min, and subsequently cooled to room temperature under running water. The absorbance of the sugar extract was measured at 625 nm, and that of the starch extract at 620 nm. The concentrations of sugar and starch were calculated by comparing the measured absorbance values with their respective standard curves (Figure A1).

### 2.6. Determination of Tamarind Polysaccharides

Tamarind polysaccharide was extracted from seeds using an organic acid precipitation method. The seeds were ground into a fine powder and sieved through a 40-mesh screen. Accurately, 0.5 g of the seed powder was weighed and mixed with 30 volumes of hot water. The pH of the suspension was adjusted to ~4.5 using citric acid. The mixture was then boiled with continuous stirring for 30 min, producing a viscous slurry. This slurry was diluted with an equal volume of 50% hot water, left to stand for 24 h, and the supernatant was extracted. A small amount of sodium hydroxide solution was added to adjust the pH to neutrality. The solution was subsequently heated until it reached a semi-fluid state, after which heating was stopped. Finally, a minimal volume of sodium bicarbonate solution was introduced, and the preparation was freeze-dried and weighed to obtain the tamarind polysaccharide.

### 2.7. Determination of Titratable Acidity

Zhidong Li revealed that tartaric acid accumulates at high concentrations in tamarind fruit and is the major contributor to its sour flavor [34]. The total acid content in tamarind fruit pulp was quantified using an acid–base titration method. The primary organic acid present in the pulp is tartaric acid; therefore, the total acid content was calculated based on the relative molecular weight and the number of protons contributed by tartaric acid. Precisely, 0.1 g of fruit pulp was weighed and transferred to a mortar. An aliquot of 4 mL of deionized water was added, and the sample was thoroughly ground to prepare a homogenate. The homogenate was centrifuged at 10,000 RPM for 10 min at 25 °C. The collected supernatant was transferred to a 5 mL volumetric flask and brought to volume with deionized water. Next, 1 mL of the test solution was transferred to a 10 mL volumetric flask and brought to volume with deionized water. Two drops of phenolphthalein ethanol solution were added as an indicator. The solution was titrated with 0.1 mol/L sodium hydroxide until a faint pink color appeared and persisted for at least 30 s. The volume of sodium hydroxide consumed (V, mL) was recorded. The tartaric acid content was calculated based on its molecular weight (150.09 g/mol) and the number of ionizable protons. The total acid content was approximated from the titration results.

One-way analysis of variance (ANOVA) was performed using SPSS 26 software, and Duncan’s multiple range test was adopted for multiple comparisons among groups. To control the false positive rate caused by multiple testing, all *p*-values obtained from correlation analysis and group comparisons were adjusted using the Benjamini–Hochberg false discovery rate (FDR) method. Adjusted *p*-values were used as the criterion for statistical significance.

### 2.8. Data Processing

#### 2.8.1. Analysis of Variance and Multiple Comparisons

All statistical analyses were performed using SPSS 26.0 software. One-way analysis of variance (ANOVA) was conducted to evaluate significant differences in phenotypic, physiological, and microbial indices among different tamarind provenances. Duncan’s multiple range test was used for post hoc multiple comparisons between groups. The Benjamini–Hochberg false discovery rate (FDR) correction was applied to adjust *p*-values for multiple testing, in order to control the false positive rate. A *p*-value < 0.05 was considered statistically significant, and *p* < 0.01 was regarded as highly significant.

#### 2.8.2. Alpha and Beta Diversity Analysis of Microbial Communities

Alpha diversity indices, including Chao1, Shannon, Simpson, and Observed_species, were calculated to assess the richness and diversity of microbial communities. Rarefaction analysis was performed with the maximum rarefaction depth set to 95% of the sequence count in the sample with the lowest sequencing depth. For beta diversity analysis, the Bray–Curtis distance matrix was calculated. Principal Coordinate Analysis (PCoA) and Unweighted Pair Group Method with Arithmetic Mean (UPGMA) clustering were performed to visualize community differences across samples. Permutational Multivariate Analysis of Variance (PERMANOVA, 999 permutations) was used to test the significance of community differentiation, with R^2^ and *p*-values reported. All diversity analyses were conducted in R software (v.4.3.3) using the vegan, ggplot2, and ggtree packages.

#### 2.8.3. Correlation Analysis Between Microbial Communities and Plant Traits

The relative abundance of each microbial phylum and all plant trait indices were subjected to standardization to a mean of zero (Z-score normalization) to unify data dimensions. Pearson correlation analysis was then performed to examine the relationships between microbial phyla and plant traits. Correlation analysis and visualization of the correlation heatmap were conducted using the Correlation Plot plugin in Origin 2021 software. Statistical significance was defined at *p* < 0.05.

## 3. Results

### 3.1. Variance Analysis of Seed and Fruit Morphology

We determined the agronomic parameters of the tested tamarind accessions, including fruit length, width, thickness, total weight, shell weight, flesh weight, number of seeds, seed weight, and silk weight. Additionally, seed length, width, thickness, and 100-seed weight were also recorded (Table A4).

Based on Table A1, the fruit morphology indices showed a minimum length of 3 cm and a maximum of 23.2 cm, indicating a wide variation range with substantial differences (coefficient of variation, CV = 0.37). Fruit weight (CV = 0.49), flesh weight (CV = 0.48), shell weight (CV = 0.54), silk weight (CV = 0.7), and seed count (CV = 0.47) all exhibited marked variability and a notable degree of dispersion. Conversely, the variation in fruit width (CV = 0.14) and thickness (CV = 0.13) was relatively moderate. With respect to seed morphology, the maximum 100-seed weight reached 71.9 g, while the minimum 100-seed weight was 24.1 g, reflecting significant variation (CV = 0.24). Among all indices, silk weight displayed the largest fluctuation range, whereas seed length (CV = 0.09) demonstrated the smallest variation.

### 3.2. Venn and Cluster Analysis of Phyllosphere and Rhizosphere Microbial

**Figure 2:**
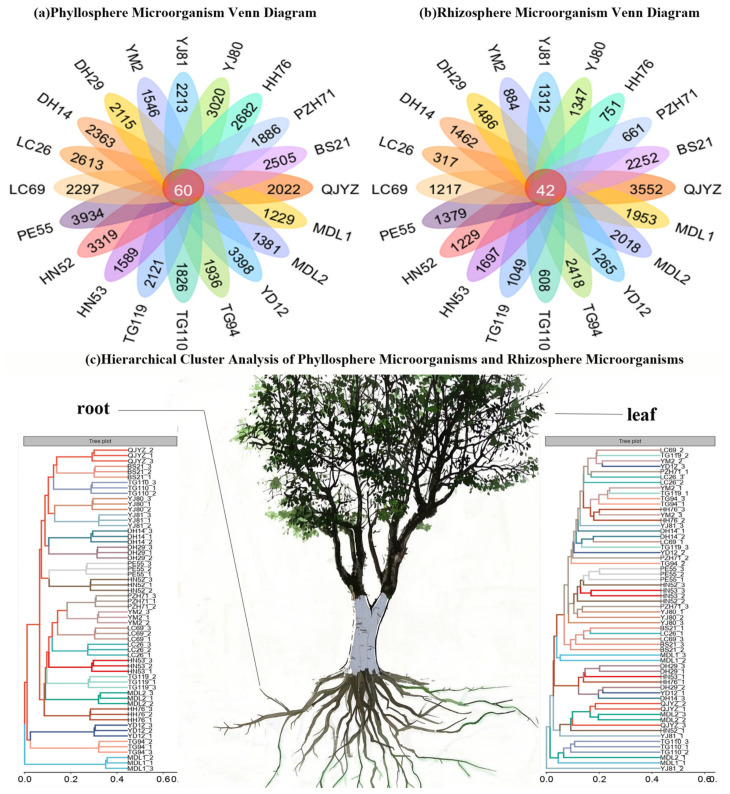
Hierarchical clustering and venn diagram analyses of microbial communities in rhizosphere and leaf samples. (**a**) The number of ASVs shared by phyllosphere bacterial communities and the number of ASVs unique to each sample. (**b**) The number of ASVs shared by phyllosphere bacterial communities and the number of ASVs unique to each sample. (**c**) Hierarchical clustering analysis of rhizosphere and leaf-associated microbial communities was performed.

**Figure 3:**
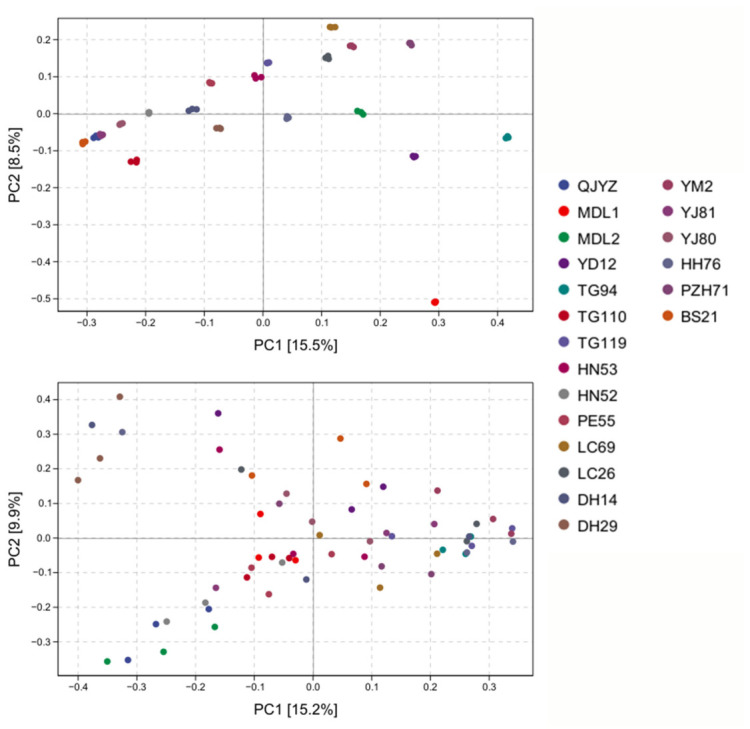
PCoA Plots of Rhizosphere Bacterial Communities (**Top**) and Phyllosphere Bacterial Communities (**Bottom**).

In this study, for 20 tamarind geographical provenances, the number of ASVs detected on leaf surfaces—which are more susceptible to insect damage and air convection than roots—was significantly higher than that detected in rhizosphere soil. However, the number of shared ASVs common across rhizosphere soil samples from different provenances exceeded that observed on leaf surfaces [35,36,37]. Microbial hierarchical clustering analysis revealed distinct community structures between leaves and roots, and the length of the clustering branches reflected significant microbial diversification across provenances (Figure 2). As depicted in Figure 3, PCoA ordination of rhizosphere and phyllosphere microbial communities (top: rhizosphere; bottom: phyllosphere) revealed divergent sample clustering patterns. Specifically, rhizosphere communities showed greater inter-group variability, which implies a lower level of overall heterogeneity in phyllosphere microbial assemblages.

### 3.3. PCA of Phyllosphere and Rhizosphere Bacterial Communities

Figure 3 consists of two Principal Coordinate Analysis (PCoA) plots based on the β-diversity distance matrix: the upper panel shows the sample distribution of rhizosphere microbial communities, and the lower panel shows that of phyllosphere microbial communities. Each point in the plots represents one sample, with different colors corresponding to different clonal groups. The percentages labeled on the axes indicate the proportion of total sample variation explained by each axis. For the rhizosphere microbial communities, the samples exhibit a wide distribution range: PC1 (15.5%) ranges from −0.3 to 0.4, and PC2 (8.5%) ranges from −0.5 to 0.2, with intra-group replicates distributed more compactly. For the phyllosphere microbial communities, PC1 (15.2%) ranges from −0.4 to 0.3, and PC2 (9.9%) ranges from −0.3 to 0.4, with intra-group replicates distributed more dispersedly.

### 3.4. Comparison Between Rhizosphere and Phyllosphere Bacterial Communities Based on Selected Plant Traits

**Figure 4:**
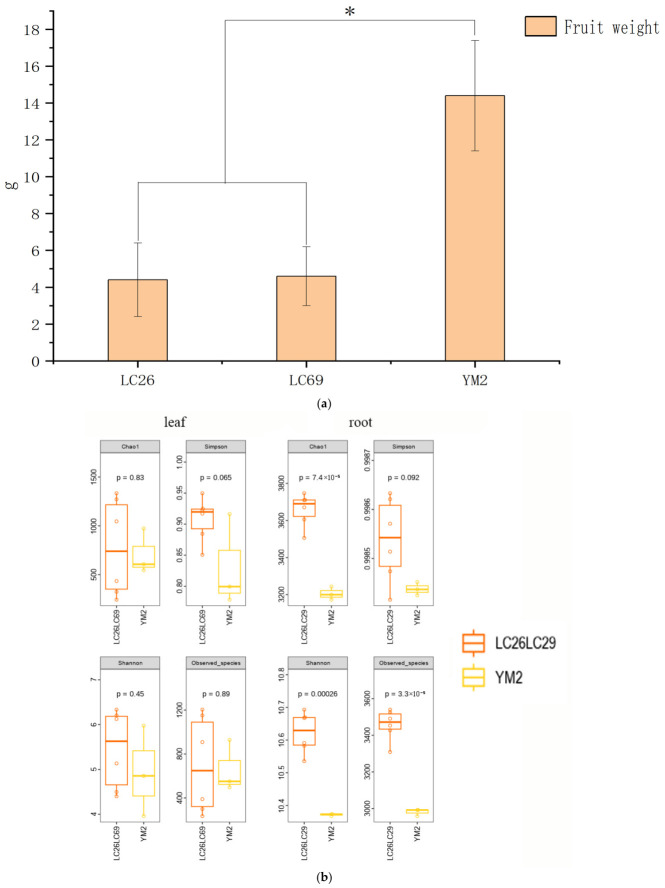

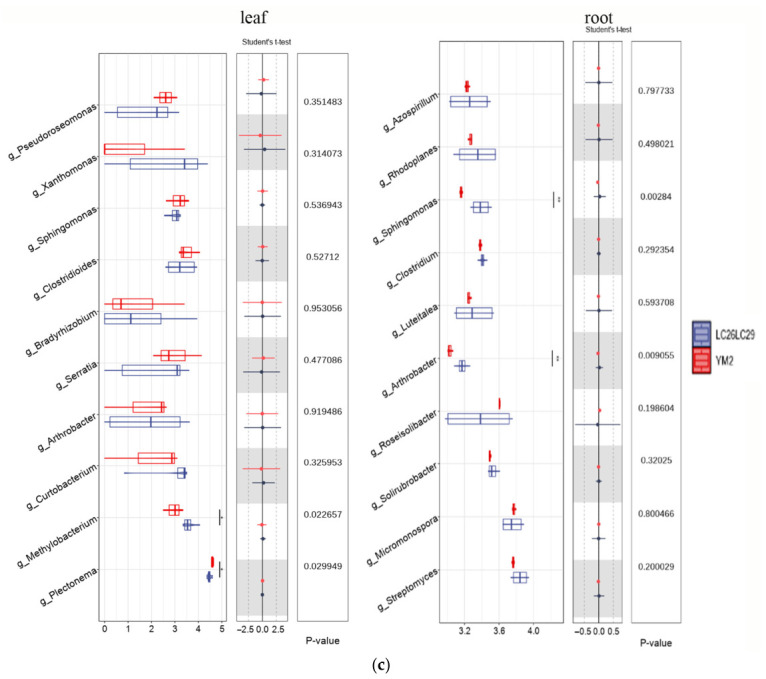
(**a**) Significant Differences in Fruit Weight Among LC26, LC69 and YM2. (**b**) Differences in α-Diversity Among LC26, LC69 and YM2. (**c**) Microbial Community Differences Between LC26, LC69 and YM2. Asterisk * indicates statistical significance at *p* < 0.05 and ** at *p* < 0.01.

Principal component analysis of fruit and seed morphological indicators was performed using SPSS 26. The maximum communality variance of fruit weight after extraction was 0.97 (Table A5), indicating that fruit weight was strongly associated with other variables and contributed most to the shared variance. Accordingly, microbial diversity analyses were conducted based on fruit weight groups. Fruit weight varied significantly across geographical regions. YM2 from Yuanmou County had the highest fruit weight, with a mean of 14.4 ± 3.0 g. In contrast, LC26 and LC69 from Lincang County had the lowest mean fruit weights, 4.4 ± 2.0 g and 4.6 ± 1.6 g, respectively. Analysis of variance (ANOVA) revealed significant differences among the groups (*p* < 0.05) (Figure 4a). Alpha diversity analysis revealed significant variation in rhizosphere microbial communities. Specifically, the Shannon index exhibited significant differences among rhizosphere bacterial communities, while the Chao1, Shannon, and observed species indices all displayed highly significant differences (Figure 4b). These findings suggest that rhizosphere bacterial communities more strongly influence tamarind fruit traits than foliar bacterial communities. Significant differences were also observed between methylobacterium and plectonema among phyllosphere bacterial communities. The abundance of Methylobacterium was lower on YM2 leaf surfaces compared to LC26 and LC69, whereas the abundance of plectonema was higher on YM2 leaf surfaces than on those of LC26 and LC69. Within rhizosphere microbial communities, significant differences were observed in the relative abundances of Sphingomonas and Arthrobacter. In YM2, both genera were present at significantly lower levels compared to LC26 and LC69.

**Figure 5:**
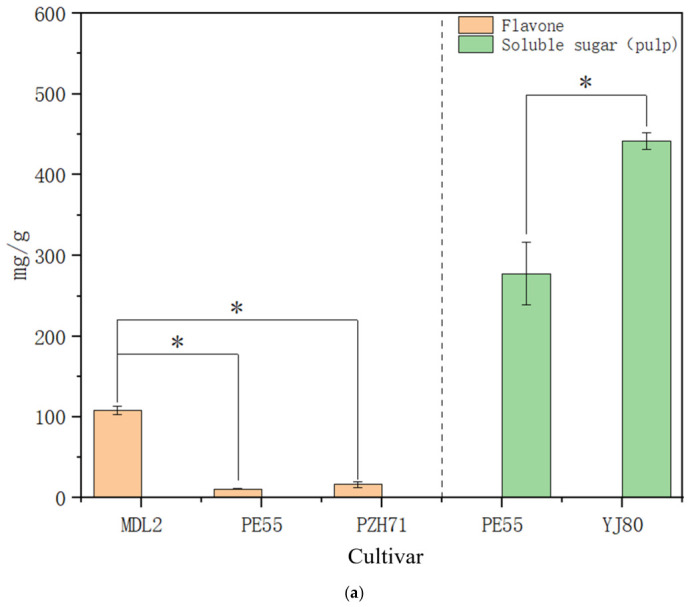

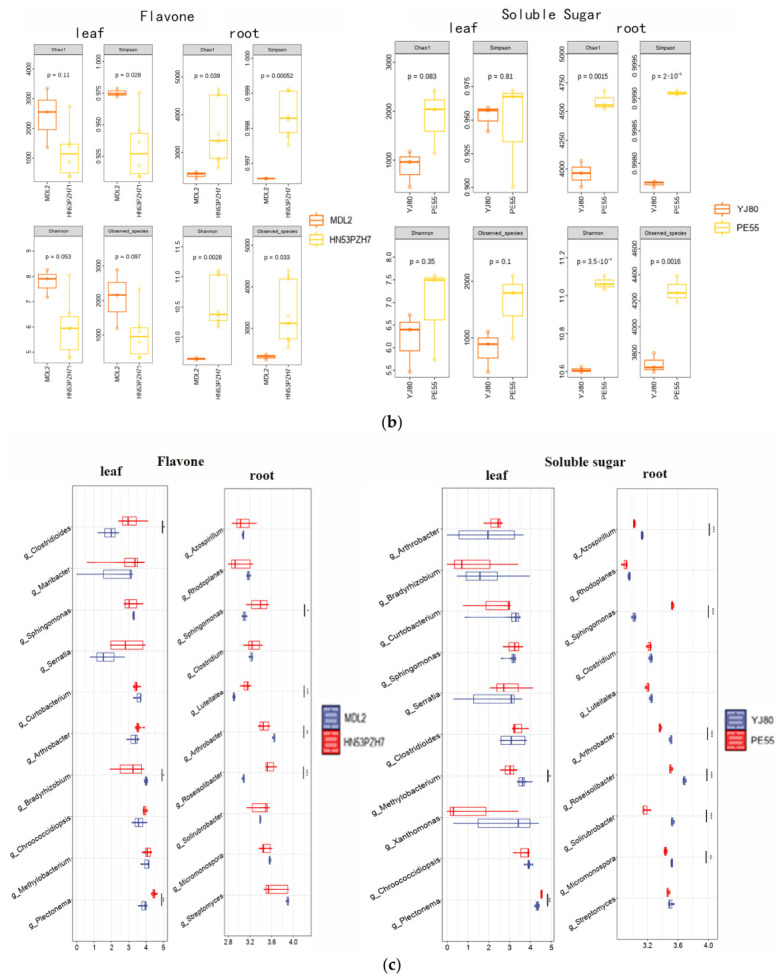
(**a**) Significant Differences in Flavone Among PZH71, PE55, HN53 and MDL2. Significant Differences in Soluble sugar in leaf Among PE55 and YJ80. (**b**) Differences in α-Diversity Among PZH71, PE55, HN53 and MDL2. Differences in α-Diversity Among PE55 and YJ80. (**c**) Microbial Community Differences Between PZH71, PE55, HN53 and MDL2. Microbial Community Differences Between PE55 and YJ80. Asterisk * indicates statistical significance at *p* < 0.05, ** at *p* < 0.01, and *** at *p* < 0.001.

Principal component analysis (PCA) was conducted on nine agronomic and physiological indicators using SPSS 26 software (Table A6). Among them, flavonoid contents exhibited the highest common factor variance (0.924; see Table A7), indicating strong associations with other variables and the greatest contribution to shared variance. Since tamarind’s primary value lies in its fruits, and fruit sugar content represents a key determinant of flavor, we investigated differences in microbial diversity in the fruit pulp with respect to flavonoids and soluble sugars. Flavonoids, a class of natural bioactive compounds characterized by a chromone ring structure, are widely distributed in plants and demonstrate diverse biological activities, including antioxidant and anti-inflammatory effects [38,39]. Among the samples, the highest flavonoid content was detected in MDL2 (Maoduoli No. 2), at 107.46 ± 5.25 mg/g. In contrast, the lowest contents were observed in PE55 from Pu’er City (11.01 ± 0.82 mg/g), PZH71 from Panzhihua City (16.41 ± 3.54 mg/g), and HN53 from Hainan Province (16.71 ± 7.40 mg/g). Regarding soluble sugars in fruit pulp, the highest content was found in YJ80 from Yuanjiang County, at 441.74 ± 10.53 mg/g, whereas the lowest was observed in PE55 from Pu’er City (277.69 ± 38.85 mg/g) (Figure 5a). Alpha diversity analysis revealed no significant variations (*p* > 0.05) in leaf-associated microbial diversity, irrespective of variation in flavonoid or sugar content in the fruit. However, for the rhizosphere microbiota, significant differences (*p* < 0.01) in alpha diversity were observed among groups grouped according to fruit pulp flavonoid and soluble sugar levels. The Simpson and Shannon indices of groups with varying flavonoid contents or fruit pulp sugar concentrations showed highly significant variations (*p* < 0.01), and all four alpha diversity indices of groups differing in fruit pulp sugar content also exhibited highly significant differences (*p* < 0.01) (Figure 5b). These results indicate that physiological variation in tamarind greatly influences rhizosphere microbial diversity than on that of phyllosphere bacterial communities. We further analyzed the rhizosphere microbiota of MDL2, PZH71, PE55, and HN53. Notably, the rhizosphere microbiota of MDL2 differed significantly from that of PZH71, PE55, and HN53 with respect to specific microbial taxa. The relative abundances of Sphingomonas, Luteitalea, and Roweisolibacter were lower in MDL2 than in PZH71, PE55, and HN53, whereas the abundance of Archaebacter was higher in MDL2. Based on correlation analysis, we hypothesize that differences in flavonoid profiles among these provenances may have contributed to the distinct rhizosphere microbiota compositions. When grouped by fruit pulp sugar content, the rhizosphere microbiota of YJ80 displayed significantly higher relative abundances of Azospirillum, Arthrobacter, Roseisolibacter, Solirubrobacter, and Micromonospora compared with PE55. Conversely, the abundance of Sphingomonas in YJ80 was significantly lower than in PE55. Previous studies have demonstrated that the genus Azospirillum possesses plant growth-promoting potential. In the present study, the abundance of this genus was positively correlated with fruit sugar content, suggesting a possible correlation with sugar accumulation, though causality cannot be inferred from the current data. However, whether this association reflects a direct regulatory effect requires further verification through inoculation experiments [39,40].

**Figure 6:**
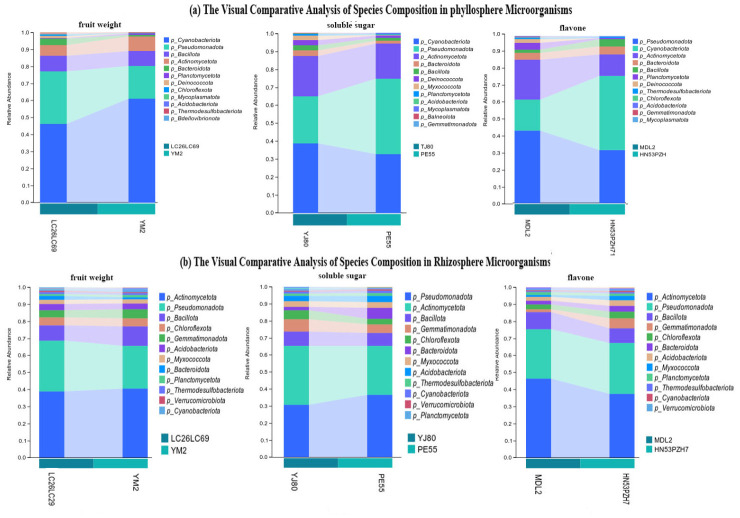
Comparative visualization of species composition in phyllosphere and rhizosphere microorganisms. (**a**) Phyllosphere Microbial Community Differences Based on Differences in Flavonoids, Fruit Weight, and Soluble Sugar (in leaf). (**b**) Rhizosphere Microbial Community Differences Based on Differences in Flavonoids, Fruit Weight, and Soluble Sugar (in leaf).

The graph provides a visual comparison of microbial composition and abundance across different indicators (Figure 6). Differences were observed in microbial community composition among the samples. In the phyllosphere microbial community, Cyanobacteria and Pseudomonas were most abundant, whereas in the rhizosphere microbial community, Actinomycetota and Pseudomonas predominated. Indicator-based groupings revealed distinct microbial community shifts. In the fruit weight-differentiated groupings, the phyllosphere microbial community of YM2, which exhibited larger fruit weight, showed higher abundances of Cyanobacteria and Bacillota but lower abundances of Pseudomonas and Bacteroidota. In groups stratified by fruit weight, no significant differences were observed in the phyllosphere microbial communities between the maximum and minimum fruit weight groups, whereas distinct differences were detected in the rhizosphere microbial communities. In the rhizosphere microbial community of YM2, no significant differences were observed in the phyllosphere microbial communities, whereas distinct differences were detected in the rhizosphere microbial communities. Actinomycetota and Bacillota abundances slightly increased, whereas Pseudomonas abundance decreased (Figure 4). In groups differentiated by fruit pulp soluble sugar content, the leaf-associated microbiota of YJ80 exhibited elevated abundances of Cyanobacteria and Bacteroidota, coupled with a reduced abundance of Pseudomonas. In the rhizosphere microbial community of YJ80, a marginal increase in Actinomycetota abundance was accompanied by a decrease in Bacillota abundance. In groupings based on leaf flavonoid content, the leaf-associated microbiota of MDL2, which contained higher flavonoid levels, showed elevated abundances of Pseudomonadota, Planctomycetota, and Actinomycetota, whereas Cyanobacteriota and Bacilota abundances were lower. In the rhizosphere microbial community of MDL2, Actinomycetota abundance was higher, while Gemmatimonadota abundance was reduced (Figure 5). Although we observed an apparent association between plant traits and symbiotic microorganisms, the lack of determination of relevant phytohormones meant that we could only postulate the existence of such an association, with no direct evidence to confirm this relationship.

### 3.5. Cluster Analysis of Fruit Morphology

**Figure 7:**
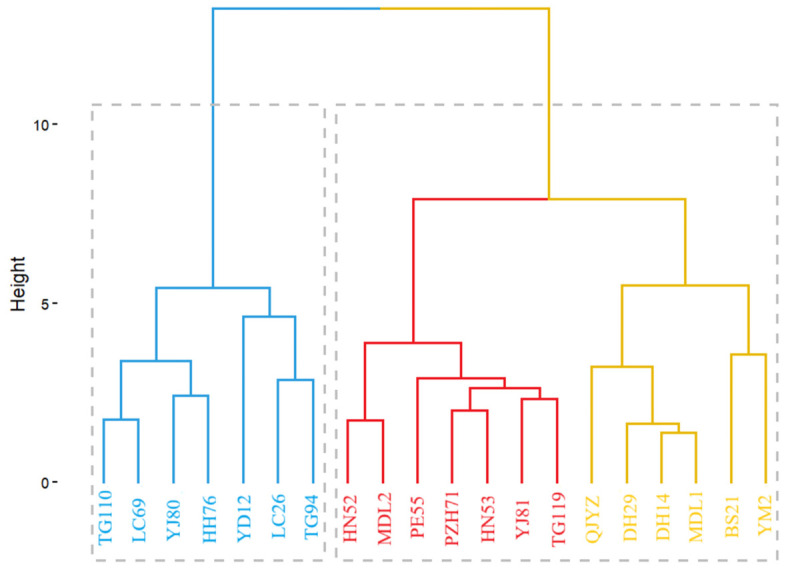
Cluster Analysis of Fruit Morphology. The dashed box indicates that the fruits of 20 geographical Tamarindus indica provenances were divided into two major populations at a clustering distance of height = 10. The red, yellow, and blue color classifications reflect the approximate division into three populations at height = 7. Specifically, blue represents the small-fruited population, red represents the medium-fruited population, and yellow represents the large-fruited population.

Cluster analysis by Euclidean distance was performed on the obtained fruit morphological data (Figure 7). It was found that the samples could be roughly divided into two groups at a clustering distance of 10: the large-fruit group and the small-fruit group. Further, the initially identified large-fruit group could be split into two subgroups at a clustering distance of 6: the medium-fruit subgroup and the large-fruit subgroup. The small-fruit group includes TG110, LC69, Y180, HH76, YD12, LC26, and TG94. The medium-fruit subgroup consists of HN52, MDL2, PE55, PZH71, HN53, and YJ81. The large-fruit subgroup comprises TG119, QY2, DH29, DH14, MDL1, BS21, and YM2. Among these, YM2 and BS21 have the largest fruits within the large-fruit subgroup.

### 3.6. Correlation Analysis Between Bacterial Communities and Plant Traits

**Figure 8:**
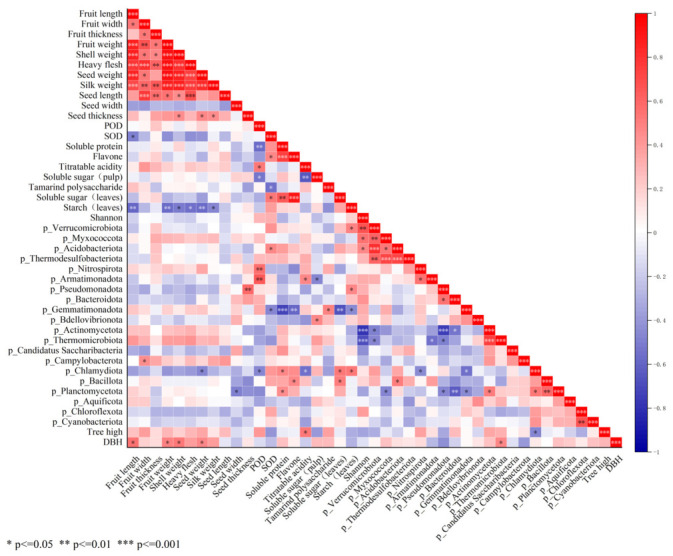
Correlation Heatmap Analysis of Various Standardized Indicators. Red indicates a positive correlation, while blue indicates a negative correlation. The darker the red color, the stronger the positive correlation; the darker the blue color, the stronger the negative correlation.

The morphological indicators of fruits and seeds, physiological and biochemical indicators, Shannon index of rhizosphere microbial communities, and relative abundances of major microbial phyla were subjected to Z-score standardization, so that they could be used for Pearson correlation analysis in the same dimension (Figure 8). Correlation analysis and visualization were performed using the Correlation Plot plugin in Origin, which does not involve PERMANOVA testing. Figure 8 shows the correlation between rhizosphere microbial communities and plant traits. In contrast, seed width, fruit width, and seed count exhibited inverse relationships with high statistical significance. Among physiological indicators, soluble sugars, soluble proteins, and flavonoids in leaves showed highly significant positive correlations. Likewise, titratable acidity in pulp and leaf POD activity were strongly and positively correlated. Tree height showed a positive correlation with titratable acidity; DBH (diameter at breast height) was positively correlated with fruit length, fruit weight, pericarp weight, and seed weight. By contrast, titratable acidity and soluble sugar content in fruit flesh were highly significantly and negatively correlated. The Shannon index of the entire rhizosphere microbial community shows a significant negative correlation with the relative abundances of p_Acidobacteriota and p_Thermodesulfobacteriota, as well as with p_Verrucomicrobiota, p_Myxococcota and p_Acidobacteriota. Various microbial phyla may show positive or negative correlations due to symbiotic relationships or antagonistic effects between them. For instance, p_Thermodesulfobacteriota shows a significant positive correlation with p_Verrucomicrobiota, p_Myxococcota, and p_Acidobacteriota, while p_Pseudomonadota exhibits a significant negative correlation with p_Actinomycetota. What is most interesting is the relationship between microbial communities and plant phenotypes. p_Planctomycetota shows a negative correlation with seed width, while exhibiting a positive correlation with soluble protein in leaves. p_Bacillota is positively correlated with flavonoids and soluble sugars in leaves. p_Chlamydiota presents a negative correlation with seed weight, peroxidase (POD), and titratable acidity in pulp, but a positive correlation with soluble protein, soluble sugar, and starch in leaves. p_Campylobacterota has a positive correlation with fruit width. p_Bdellovibrionota is positively correlated with soluble sugar in pulp. p_Gemmatimonadota shows negative correlations with superoxide dismutase (SOD), soluble protein, flavonoids, soluble sugar, and starch in leaves. p_Pseudomonadota is positively correlated with seed thickness and starch in leaves. p_Armatimonadota exhibits a positive correlation with POD and titratable acidity in pulp, but a negative correlation with sugar in pulp. p_Nitrospirota is positively correlated with POD, and p_Acidobacteriota shows a positive correlation with SOD.

## 4. Discussion

This study aims to investigate the relationship between rhizosphere bacterial communities and the traits of tamarind. Under the uniform field management conditions of soil loosening, weeding, and irrigation provided by Maoduoli Manor, different tamarind geographical provenances exhibited significant morphological and physiological variation. Evaluation of 20 provenances revealed distinct traits: BS21 from Baoshan County and YM2 from Yuanmou County produced the largest fruits; MDL2 leaves contained the highest flavonoid content; YJ80 from Yuanjiang County had the highest fruit sugar content; BS21 from Baoshan City showed the highest soluble protein content in leaves; TG119 from Thailand exhibited the highest titratable acidity in pulp; and HN53 from Hainan Province contained the highest tamarind polysaccharide content. These findings provide an important basis for selecting superior provenances in future breeding programs.

To identify the microbial taxa exerting the greatest influence on plant traits, using soluble sugars, flavonoids, and fruit weight as comparative indicators. Comparative analysis revealed that significant differences were observed among individual samples of the rhizosphere microbiota, no significant differences were observed among phyllosphere microbial samples under these traits, and the diversity of phyllosphere microbiota was lower than that of rhizosphere microbiota. This finding is consistent with the findings by Guangfei Wei [41]. Studies indicate that host plants, alongside environmental factors, are critical determinants of Methylobacterium community composition [42]. Methylobacterium species can metabolize plant-derived methanol and are able to synthesize auxin and cytokinin [43,44,45]. The abundance of Methylobacterium in the phyllosphere of LC26 and LC69 was relatively high, and these two provenances exhibited lower fruit weights. Combined with the well-documented trait of Methylobacterium to synthesize auxins and cytokinins in previous studies, it is speculated that the abundance of this genus may have a potential association with fruit development. However, this remains speculative and requires functional validation. In contrast, plectonema abundance was significantly higher on YM2 leaves than on LC26 and LC29 leaves (*p* < 0.05). This observation aligns with previous findings showing that cyanobacteria can positively influence plant growth and development [46]. With the exception that significant differences in rhizosphere microbial communities were detected among geographical provenances of tamarind with substantial variations in individual traits, correlation analysis indicated that various phenotypic traits of tamarind exhibited a certain degree of correlation with the relative abundances of different bacterial phyla under these grafting conditions. Rodríguez [47] noted that genetic factors can influence plant rhizosphere microbiomes. The association between rhizosphere microbiomes and geographical provenances observed in this study may reflect the indirect regulation of the rhizosphere microenvironment by scion traits. However, due to the constraints of the grafting design, the independent effects of scions and rootstocks cannot be distinguished at present. Among the traits of tamarind found to be correlated with various bacterial phyla, Bacillota exhibited a positive correlation with flavonoid contents in tamarind leaves. The phylum also showed a positive correlation with flavonoid and soluble sugar contents in leaves. This finding is consistent with the report by Abdelkhalek [48], wherein *Bacillus licheniformis* strain POT1 significantly increased flavonoid levels in *Solanum tuberosum*. These results suggest that bacterial communities within this phylum showed correlation with plant secondary metabolites; however, only an associative relationship was observed in the present study, and a causal effect cannot be confirmed at this stage. p_Verrucomicrobiota exhibits a positive correlation with starch content in leaves. However, to date, the only documented research on strains within Verrucomicrobiota has focused on their ability to promote root growth in rice [48]. P-Acidobacteriota exhibits a positive correlation with leaf SOD activity. Consistent with previous findings, Acidobacteriota is known to possess functionalities including nitrogen fixation, phosphate solubilization, extracellular polysaccharide (EPS) production, siderophore biosynthesis, and plant growth hormone secretion, as well as harboring genes involved in the regulation of oxidative stress [49]. p_Pseudomonadota is one of the most extensively studied bacterial phyla; within this phylum, rhizobia act as critical contributors to plant stress tolerance and nitrogen fixation [49]. In the present study, p_Pseudomonadota exhibited a positive correlation with seed width and starch content in leaves under these grafting conditions. Although previous studies have documented plant–microbiome interactions, the grafting design employed in this study precluded the attribution of rhizosphere microbiome differences solely to scion genotype. Furthermore, the absence of plant hormone quantification data prevented the elucidation of the regulatory pathways involving scions, rootstocks, and bacterial communities. Therefore, the conclusions of this study are limited to the specific grafting conditions described herein and reflect only associative, rather than causal, relationships.

We recognize that the use of rootstocks from the same source without ensuring genotypic consistency represents an experimental trade-off. In practice, however, rooted cuttings or clonal seedlings are rarely used as rootstocks because such asexually propagated plants often have underdeveloped root systems that do not meet the requirements for suitable rootstocks. Even in this study, using seedling rootstocks did not achieve a 100% survival rate, which resulted in a random spatial distribution of the grafted plants used in the experiment. These uncontrollable factors prevent us from distinguishing the independent effects of scion genotype, rootstock genotype, and their interaction. Therefore, the observed associations between rhizosphere microbiome and plant traits may reflect both the direct influence of scion genetics on root exudation and the interactive effects between scion and rootstock. In addition, this study only analyzed bacterial communities and did not include fungi or other microorganisms, which may also play important roles in plant growth and adaptation. Furthermore, the study relied on 16S rRNA gene sequencing rather than shotgun metagenomics, which limited our understanding of microbial functions. These limitations should be considered and improved upon in future research.

## 5. Conclusions

This study analyzed the diversity of rhizosphere and phyllosphere microbial communities among 20 tamarind provenances and their associations with plant agronomic and physiological traits. The results showed that rhizosphere microbial communities exhibited greater diversity and stronger associations with key traits than phyllosphere microbiota. Although the graft-based experimental design limits the distinction between scion and rootstock effects, this study provides preliminary evidence that tamarind rhizosphere microbial communities are associated with scion geographical provenances. These findings lay a foundation for further exploring the mechanism of plant–microbe interactions in tamarind and developing targeted microbial inoculants.

## Figures and Tables

**Table 1:** Examples of plant–microbe interactions in previous studies.

Study	Result
Reena J. and Bagyaraj (1990) [5]	It was found that VAM fungi has a positive impact on the growth of tamarind [5].
Shapiro L. (2012) [6]	*Erwinia tracheiphila* enhances beetle vector attraction to promote its own transmission by modifying volatile compounds in the leaves and flowers of *Cucurbita pepo* ssp. *texana*, whereas Zucchini yellow mosaic virus suppresses floral volatile emissions to inhibit beetle aggregation, thereby reducing the likelihood of the host exposure to the former [6].
Innerebner, G. (2011) [7]	In a controlled model system, plant-associated *Sphingomonas* strains can significantly inhibit the proliferation of leaf-pathogenic *Pseudomonas syringae* pv. tomato DC3000 and *Xanthomonas campestris* pv. *campestris* on *Arabidopsis thaliana* and mitigate disease symptoms through mechanisms such as modifying carbon source utilization [7].
Ritpitakphong U. (2016) [8]	The microbiome present on the leaf surface of *Arabidopsis* protects against fungal pathogens [8].
Tuo L. (2025) [9]	By knocking out the *TgMFS4* gene in *Trichoderma guizhouense*, the experiment converted the previously antagonistic interaction between *Bacillus velezensis* and *T. guizhouense* into a cooperative relationship, which elevated their colonization levels in the tomato rhizosphere and significantly improved the control efficacy against *Fusarium* wilt disease [9].
Yaowei C. (2025) [10]	The plant growth-promoting rhizobacteria (PGPR) *Bacillus megaterium* NCT-2 facilitates cadmium phytoremediation by modulating the rhizosphere nutrient microenvironment and activating amino acid metabolic and synthetic pathways in the rhizosphere [10].
Ahsan T. (2024) [11]	The combined application of microbial fertilizer, compound fertilizer, and microbial agent can substantially enhance peanut growth vigor and boost yield [11].

**Table 2:** Origins of 20 geographical provenances of tamarind.

Geographical Provenances	Administrative Area	Distributed Watershed	Remark
HN52	Hainan province		
HN53	Hainan province		
PZH71	Panzhihua City, Sichuan Province		
YM2	yuanmou County, Yunnan Province	Jinsa River Basin	
MDL1	Pu’er City, Yunnan Province	Lancang River Basin	Maoduoli Group cultivates
PE55	Pu’er City, Yunnan Province	Lancang River Basin	
LC69	Lincang City, Yunnan Province	Lancang River Basin	
LC26	Lincang City, Yunnan Province	Lancang River Basin	
HH76	Honghe Prefecture, Yunnan Province	Honghe River Basin	
YJ81	Yuanjiang County, Yunnan Province	Honghe River Basin	
YJ80	Yuanjiang County, Yunnan Province	Honghe River Basin	
BS21	Baoshan City, YunnanProvince	Nujiang River Basin	
DH29	Dehong Prefecture, Yunnan province	Nujiang River Basin	
DH14	Dehong Prefecture, Yunnan province	Nujiang River Basin	
TG119	Thailand		
TG110	Thailand		
TG94	Thailand		
TD12	India		
QJYZ	Pu’er City, Yunnan Province		
MDL2			Maoduoli Group cultivates

## Data Availability

The raw sequencing data have been deposited in the NCBI Sequence Read Archive (SRA) under the accession number PRJNA1283163.

## Appendix A

**Table A1:** PCR reaction system.

Component	Volume (μL)
Abclonal DNA polymerase	0.25
5× Reaction buffer	5
5× High GC buffer	5
dNTP (10 mM)	2
Template DNA	2
Forward primer (338F, 10 μm)	1
Reverse primer (806R, 10 μm)	1
Water	8.75
Total volume	25

PCR cycling conditions: pre-denaturation at 95 °C for 5 min; 35 cycles of denaturation at 95 °C for 30 s, annealing at 55 °C for 30 s, and extension at 72 °C for 45 s; final extension at 72 °C for 10 min.

**Table A2:** Statistics of Sequencing Reads Before and After Denoising for Rhizosphere Microbiota.

Sample ID	Input	Filtered	Denoised	Merged	Non-Chimeric	Non-Singleton	Retention Rate	Denoising Efficiency	Chimera Removal Rate	Final Effective Rate
QJYZ_1	99,962	80,780	76,390	56,300	53,829	53,485	0.81	0.95	0.04	0.54
QJYZ_2	103,108	83,337	78,836	59,766	57,351	57,014	0.81	0.95	0.04	0.55
QJYZ_3	101,897	82,168	78,370	62,898	60,957	60,651	0.81	0.95	0.03	0.6
MDL1_1	104,771	85,671	83,721	75,532	73,361	73,265	0.82	0.98	0.03	0.7
MDL1_2	101,843	83,508	81,782	73,616	71,495	71,421	0.82	0.98	0.03	0.7
MDL1_3	98,836	81,554	79,880	73,679	72,202	72,141	0.83	0.98	0.02	0.73
MDL2_1	95,711	78,998	75,909	62,249	60,197	60,048	0.83	0.96	0.03	0.63
MDL2_2	105,365	85,921	82,741	68,571	66,279	66,115	0.82	0.96	0.03	0.63
MDL2_3	106,259	87,194	83,743	68,409	66,105	65,917	0.82	0.96	0.03	0.62
YD12_1	101,380	82,520	77,276	55,652	50,482	49,845	0.81	0.94	0.09	0.49
YD12_2	93,117	76,207	71,561	51,973	47,774	47,211	0.82	0.94	0.08	0.51
YD12_3	107,121	86,838	81,685	60,086	54,937	54,269	0.81	0.94	0.09	0.51
TG94_1	98,291	80,616	77,863	67,823	66,774	66,641	0.82	0.97	0.02	0.68
TG94_2	95,367	78,270	74,930	61,112	59,532	59,383	0.82	0.96	0.03	0.62
TG94_3	91,801	75,686	73,017	63,209	62,288	62,161	0.82	0.96	0.01	0.68
TG110_1	92,803	74,917	71,773	60,501	58,718	58,543	0.81	0.96	0.03	0.63
TG110_2	99,942	80,917	77,676	67,136	65,611	65,443	0.81	0.96	0.02	0.65
TG110_3	96,699	78,284	75,051	64,756	63,212	63,020	0.81	0.96	0.02	0.65
TG119_1	94,711	76,775	72,214	55,017	53,282	53,013	0.81	0.94	0.03	0.56
TG119_2	94,423	75,997	71,679	54,784	53,020	52,754	0.8	0.94	0.03	0.56
TG119_3	100,455	81,303	76,791	58,699	56,808	56,532	0.81	0.94	0.03	0.56
HN53_1	96,222	78,707	75,111	60,476	58,376	58,119	0.82	0.95	0.03	0.6
HN53_2	87,035	71,151	67,504	53,230	51,162	50,911	0.82	0.95	0.04	0.58
HN53_3	98,766	80,227	76,690	63,906	62,104	61,870	0.81	0.96	0.03	0.63
HN52_1	137,109	112,248	105,017	72,962	69,090	68,444	0.82	0.94	0.05	0.5
HN52_2	134,934	110,514	104,697	78,403	74,937	74,394	0.82	0.95	0.04	0.55
HN52_3	137,374	112,490	106,601	81,724	78,276	77,722	0.82	0.95	0.04	0.57
PE55_1	134,320	108,665	102,214	73,988	69,550	68,780	0.81	0.94	0.06	0.51
PE55_2	130,319	106,157	99,359	70,640	66,421	65,649	0.81	0.94	0.06	0.5
PE55_3	134,358	109,703	102,015	69,554	65,256	64,323	0.82	0.93	0.06	0.48
LC69_1	136,617	111,708	106,787	84,593	81,762	81,413	0.82	0.96	0.03	0.6
LC69_2	144,561	117,536	112,396	90,455	87,371	87,078	0.81	0.96	0.03	0.6
LC69_3	130,126	105,794	101,115	79,824	76,954	76,629	0.81	0.96	0.04	0.59
LC26_1	136,916	112,362	108,787	96,291	94,821	94,676	0.82	0.97	0.02	0.69
LC26_2	132,829	109,282	105,771	92,956	91,334	91,173	0.82	0.97	0.02	0.69
LC26_3	137,934	112,636	109,519	98,019	96,684	96,554	0.82	0.97	0.01	0.7
DH14_1	132,684	107,915	103,574	85,918	83,639	83,414	0.81	0.96	0.03	0.63
DH14_2	139,743	113,451	109,389	94,059	92,070	91,816	0.81	0.96	0.02	0.66
DH14_3	119,688	97,579	93,910	79,798	77,988	77,769	0.82	0.96	0.02	0.65
DH29_1	141,488	113,322	108,536	87,106	84,144	83,797	0.8	0.96	0.03	0.59
DH29_2	128,708	102,725	98,560	79,255	76,548	76,249	0.8	0.96	0.03	0.59
DH29_3	133,682	106,741	102,283	82,916	80,499	80,214	0.8	0.96	0.03	0.6
YM2_1	132,267	108,752	104,354	83,247	80,767	80,527	0.82	0.96	0.03	0.61
YM2_2	135,778	111,083	106,099	81,751	78,511	78,216	0.82	0.96	0.04	0.58
YM2_3	138,647	113,559	108,695	85,067	81,927	81,679	0.82	0.96	0.04	0.59
YJ81_1	139,844	111,256	106,002	78,861	74,533	74,095	0.8	0.95	0.05	0.53
YJ81_2	138,731	110,363	105,274	79,032	74,394	73,942	0.8	0.95	0.06	0.53
YJ81_3	141,075	112,138	107,379	83,926	79,873	79,501	0.79	0.96	0.05	0.56
YJ80_1	142,145	115,027	108,476	79,493	74,837	74,256	0.81	0.94	0.06	0.52
YJ80_2	132,788	107,373	100,902	72,688	68,438	67,825	0.81	0.94	0.06	0.51
YJ80_3	132,609	107,033	101,078	73,569	69,306	68,714	0.81	0.94	0.06	0.52
HH76_1	144,040	116,874	112,760	96,165	93,568	93,302	0.81	0.96	0.03	0.65
HH76_2	136,800	111,621	107,724	92,937	90,707	90,467	0.82	0.97	0.02	0.66
HH76_3	138,024	112,547	109,091	97,017	95,164	94,941	0.82	0.97	0.02	0.69
PZH71_1	130,016	107,599	103,771	85,904	83,387	83,159	0.83	0.96	0.03	0.64
PZH71_2	131,360	107,354	103,171	86,044	83,581	83,370	0.82	0.96	0.03	0.63
PZH71_3	139,794	114,964	110,038	88,918	85,773	85,485	0.82	0.96	0.04	0.61
BS21_1	137,219	110,679	104,324	72,441	68,053	67,437	0.81	0.94	0.06	0.49
BS21_2	130,644	105,691	99,836	71,011	67,296	66,809	0.81	0.94	0.05	0.51
BS21_3	132,649	106,200	100,558	71,073	67,245	66,673	0.8	0.95	0.05	0.5

**Table A3:** Statistics of Sequencing Reads Before and After Denoising for *Phyllosphere* Microbiota.

SampleID	Input	Filtered	Denoised	Merged	Non-Chimeric	Non-Singleton	Retention Rate	Denoising Efficiency	Chimera Removal Rate	Final Effective Rate
QJYZ_1	130,974	108,927	106,569	86,538	64,530	63,086	0.83	0.98	0.25	0.48
QJYZ_2	138,087	114,913	112,614	93,701	73,219	72,267	0.83	0.98	0.22	0.52
QJYZ_3	137,856	114,399	111,308	89,001	72,352	71,604	0.83	0.97	0.19	0.52
MDL1_1	135,126	111,550	109,431	98,479	78,984	78,555	0.83	0.98	0.2	0.58
MDL1_2	142,220	118,228	116,132	102,194	81,838	81,352	0.83	0.98	0.2	0.57
MDL1_3	142,244	118,177	116,627	105,806	82,884	82,517	0.83	0.99	0.22	0.58
MDL2_1	133,869	111,927	109,295	92,255	78,687	78,250	0.84	0.98	0.15	0.58
MDL2_2	130,781	108,075	106,263	92,362	73,248	72,654	0.83	0.98	0.21	0.56
MDL2_3	130,819	108,714	107,119	90,372	69,510	68,530	0.83	0.99	0.23	0.52
YD12_1	137,877	114,407	113,340	107,250	98,547	98,406	0.83	0.99	0.08	0.71
YD12_2	130,223	107,120	105,889	99,152	82,278	82,132	0.82	0.99	0.17	0.63
YD12_3	131,737	108,289	106,964	96,335	80,879	80,218	0.82	0.99	0.16	0.61
TG94_1	139,281	115,764	114,467	107,166	93,265	92,999	0.83	0.99	0.13	0.67
TG94_2	141,427	116,983	114,080	97,132	82,652	82,167	0.83	0.98	0.15	0.58
TG94_3	136,960	113,922	112,338	101,798	93,074	92,633	0.83	0.99	0.09	0.68
TG110_1	142,415	117,137	115,633	106,888	90,318	90,168	0.82	0.99	0.16	0.63
TG110_2	141,434	114,986	113,328	106,438	90,249	90,126	0.81	0.99	0.15	0.64
TG110_3	138,778	115,118	113,907	106,666	91,340	91,212	0.83	0.99	0.14	0.66
TG119_1	133,508	111,391	109,974	102,767	92,168	92,002	0.83	0.99	0.1	0.69
TG119_2	131,321	108,501	107,127	98,833	89,434	89,132	0.83	0.99	0.1	0.68
TG119_3	138,112	114,586	113,704	107,634	89,659	89,523	0.83	0.99	0.17	0.65
HN53_1	138,256	113,944	112,532	103,003	89,616	89,366	0.82	0.99	0.13	0.65
HN53_2	129,502	107,643	106,294	96,921	82,995	82,688	0.83	0.99	0.14	0.64
HN53_3	129,058	106,671	104,959	90,155	76,065	75,361	0.83	0.98	0.16	0.58
HN52_1	110,943	85,464	84,006	74,098	58,015	57,612	0.77	0.98	0.22	0.52
HN52_2	99,529	81,770	80,545	74,423	63,709	63,516	0.82	0.99	0.14	0.64
HN52_3	98,261	77,979	76,936	67,119	53,830	53,299	0.79	0.99	0.2	0.54
PE55_1	113,679	89,240	88,352	80,824	70,068	69,847	0.79	0.99	0.13	0.61
PE55_2	111,888	89,657	88,368	77,907	64,350	63,823	0.8	0.99	0.17	0.57
PE55_3	107,699	88,349	86,864	74,700	60,666	60,058	0.82	0.98	0.19	0.56
LC69_1	105,258	86,229	85,177	77,254	63,851	63,494	0.82	0.99	0.17	0.6
LC69_2	110,178	86,409	85,444	79,502	70,119	69,933	0.78	0.99	0.12	0.63
LC69_3	106,645	87,406	86,582	78,977	65,045	64,698	0.82	0.99	0.18	0.61
LC26_1	96,059	78,987	78,312	75,334	66,742	66,671	0.82	0.99	0.11	0.69
LC26_2	94,481	78,438	77,895	75,349	64,954	64,904	0.83	0.99	0.14	0.69
LC26_3	106,026	87,169	86,474	82,728	73,807	73,734	0.82	0.99	0.11	0.7
DH14_1	115,549	94,537	92,069	79,915	69,945	69,756	0.82	0.97	0.12	0.6
DH14_2	107,937	88,356	87,267	78,881	68,683	68,508	0.82	0.99	0.13	0.63
DH14_3	91,177	74,614	73,472	64,597	56,419	55,704	0.82	0.98	0.13	0.61
DH29_1	116,606	95,153	93,589	82,782	69,125	68,789	0.82	0.98	0.16	0.59
DH29_2	101,945	82,712	81,834	76,505	72,361	72,154	0.81	0.99	0.05	0.71
DH29_3	103,407	84,025	82,399	70,134	60,574	60,092	0.81	0.98	0.14	0.58
YM2_1	91,424	73,167	72,464	68,217	63,760	63,666	0.8	0.99	0.07	0.7
YM2_2	10,2051	83,538	82,409	75,806	69,352	69,179	0.82	0.99	0.09	0.68
YM2_3	99,901	82,407	81,647	76,836	69,100	68,996	0.82	0.99	0.1	0.69
YJ81_1	106,415	85,099	83,294	69,691	56,665	56,218	0.8	0.98	0.19	0.53
YJ81_2	92,995	72,229	71,903	70,919	65,093	65,080	0.78	0.995	0.08	0.7
YJ81_3	112,423	90,631	87,663	71,038	64,302	63,940	0.81	0.97	0.09	0.57
YJ80_1	129,000	106,506	104,362	91,183	74,321	73,916	0.83	0.98	0.18	0.57
YJ80_2	130,798	105,228	103,572	94,059	76,555	76,310	0.8	0.98	0.19	0.58
YJ80_3	127,973	100,504	99,741	95,863	88,492	88,420	0.79	0.99	0.08	0.69
HH76_1	143,238	115,614	113,861	103,247	86,669	86,329	0.81	0.98	0.16	0.6
HH76_2	133,685	109,904	109,136	105,911	92,905	92,844	0.82	0.99	0.12	0.69
HH76_3	141,433	117,372	116,725	113,739	99,978	99,964	0.83	0.99	0.12	0.71
PZH71_1	137,566	108,139	107,299	103,597	91,573	91,525	0.79	0.99	0.12	0.67
PZH71_2	143,756	118,223	117,381	112,693	99,858	99,783	0.82	0.99	0.11	0.69
PZH71_3	140,119	115,737	114,377	106,128	89,475	89,286	0.83	0.99	0.16	0.64
BS21_1	129,848	107,112	104,913	93,194	82,119	81,877	0.82	0.98	0.12	0.63
BS21_2	129,962	106,289	102,995	86,450	76,587	76,103	0.82	0.97	0.11	0.59
BS21_3	140,574	113,772	110,231	90,719	80,555	80,033	0.81	0.97	0.11	0.57

**Table A4:** ANOVA of fruit and seed morphological traits.

Geographical Provenance	Fruit										Seed			
	Percent of Straight Fruit/%	Fruit Length/cm	Fruit Width/mm	Fruit Thick/mm	Fruit Weight/g	Number of Seeds	Shell Weight/g	Heavy Flesh/g	Seeds Weight/g	Silk Network Weight/g	Seed Length/mm	Seed Width/mm	Seed Thick/mm	Hundred-Grain Weight/g
HN53	10	13.6 ± 3.6a	21.3 ± 2.0bc	14.5 ± 0.9efghi	10.5 ± 3.3cde	6.5 ± 1.3bc	2.3 ± 0.5bcdef	4.4 ± 1.4bcd	3.3 ± 1.2cd	0.27 ± 0.10bcd	11.3 ± 1.11def	9.67 ± 0.3bcd	6.08 ± 0.12fgh	49.8
YM2	33.3	12.8 ± 3.1ab	23.7 ± 1.2a	16.8 ± 1.3ab	14.4 ± 3.0a	6.3 ± 2.7bc	3.0 ± 1.2ab	6.0 ± 1.2a	4.5 ± 1.3ab	0.66 ± 0.18a	14.3 ± 0.77a	10.59 ± 0.3b	6.58 ± 0.12cd	71.7
BS21	11.1	12.6 ± 3.7ab	20.1 ± 1.6cde	16.3 ± 0.9bc	14 ± 5.2ab	8.0 ± 3.8a	3.4 ± 0.7a	4.6 ± 1.7bc	5.1 ± 1.9a	0.65 ± 0.40a	11.4 ± 1.91def	9.63 ± 0.3bcd	8.18 ± 0.12a	61.9
DH14	45	11.7 ± 3.6abc	22.7 ± 2.0ab	14.9 ± 1.0defg	11.7 ± 4.1bcd	5.0 ± 1.6cdef	2.7 ± 0.8abc	5.3 ± 1.7ab	2.8 ± 1.2cdef	0.34 ± 0.25b	12.4 ± 0.89c	9.27 ± 0.3bcd	6.56 ± 0.12cd	53.5
DH29	59.1	11.4 ± 4.9abc	21.5 ± 1.9bc	15.9 ± 1.0bcd	13.5 ± 7.7abc	5.7 ± 2.0bc	2.6 ± 0.7abcd	5.7 ± 2.7a	3.7 ± 2.0bc	0.34 ± 0.21b	13.4 ± 1.39b	9.61 ± 0.3bcd	6.44 ± 0.12cdef	57.4
PZH71	46.7	11.3 ± 4.3abcd	20.6 ± 2.3cd	12.9 ± 1.7k	10.1 ± 3.3cdef	7.4 ± 2.9ab	2.2 ± 0.8bcdefg	3.9 ± 1.2cde	3.7 ± 1.3bc	0.25 ± 0.10bcde	11.7 ± 1.21def	8.78 ± 0.3def	6.22 ± 0.12defg	48.9
MDL1	53.3	11.1 ± 3.1abcde	21.3 ± 1.5bc	15.5 ± 1.2cde	12.1 ± 3.5abc	5.4 ± 0.9cde	2.6 ± 0.4abcd	5.3 ± 1.5ab	3.7 ± 1.3bc	0.34 ± 0.12b	12.5 ± 1.35c	10.58 ± 0.3b	6.71 ± 0.12c	67.5
PE55	36.7	10.7 ± 3.4bcdef	19.8 ± 1.8de	14.4 ± 1.0fghi	10.3 ± 3.9cdef	5.8 ± 2.7cd	2.2 ± 0.8cdefg	3.4 ± 1.2def	4.4 ± 1.9ab	0.29 ± 0.13bc	11.3 ± 1.43ef	10.48 ± 0.3b	7.44 ± 0.12b	71.9
YJ81	30	10.1 ± 3.6cdefg	19.5 ± 1.2def	14.2 ± 0.6fghijo	7.9 ± 3.1efghi	4.9 ± 2.0cdef	2.4 ± 0.5bcde	3.2 ± 1.2efg	2.4 ± 1.1defg	0.28 ± 0.13bcd	11.0 ± 1.07fg	9.67 ± 0.3bcd	5.74 ± 0.12hi	47.9
TG119	46.7	10.0 ± 3.4cdefg	21.3 ± 2.6bc	15.7 ± 1.2bcd	9.3 ± 2.8defg	6.1 ± 2.9bcd	2.1 ± 1.1cdefg	3.7 ± 1.3cde	3.1 ± 1.0cde	0.25 ± 0.12bcde	12 ± 1.15cde	8.8 ± 0.3def	6.57 ± 0.12cd	49.2
QJYZ	53.3	9.6 ± 2.4cdefg	22.6 ± 2.1ab	17.3 ± 1.2a	9.9 ± 2.8cdefg	4.6 ± 2.0def	2.1 ± 0.7cdefg	5.0 ± 1.5ab	2.4 ± 0.8defg	0.35 ± 0.12b	13.5 ± 1.06b	9.03 ± 0.3de	5.97 ± 0.12gh	54
MDL2	46.7	9.5 ± 3.7cdefg	22.4 ± 2.3ab	14.1 ± 1.0hijk	7.6 ± 3.6fghi	4.1 ± 1.7efgh	2.5 ± 0.4defgh	3.3 ± 1.4ef	2.1 ± 1.3fghi	0.23 ± 0.17bcde	13.3 ± 1.49b	9.65 ± 0.3bcd	5.86 ± 0.12gh	53.2
TG94	26.7	9.3 ± 3.8cdefg	18.2 ± 2.0fg	13.8 ± 1.6ghijk	5.5 ± 2.9hij	5.3 ± 2.0cde	1.6 ± 0.8efgh	2.3 ± 1.2gh	1.5 ± 1.0ghij	0.17 ± 0.10de	10.3 ± 1.36gh	7.86 ± 0.3f	5.41 ± 0.12i	30.6
YJ80	77.8	8.8 ± 2.1defgh	18.8 ± 2.6ef	14.3 ± 2.9fghij	7.5 ± 4.1ghi	5 ± 1.1cdef	1.6 ± 0.4fgh	3.4 ± 1.8def	2.3 ± 1.5efghi	0.20 ± 0.15cde	9.9 ± 1.67hi	12.04 ± 0.3a	6.48 ± 0.12cde	56.2
YD12	40	8.5 ± 2.7efghi	17.1 ± 1.5gh	14.7 ± 1.1defgh	6.1 ± 2.8hij	5.6 ± 2.0cde	1.9 ± 3.0defgh	2.6 ± 1.2fgh	1.4 ± 0.9hij	0.14 ± 0.13e	8.6 ± 0.65k	8.23 ± 0.3ef	4.88 ± 0.12j	24.1
TG110	76.7	8.3 ± 2.0fghi	16.1 ± 1.6h	13.5 ± 1.1ijk	6.3 ± 2.0hij	4.4 ± 2.4defg	1.8 ± 0.9defgh	2.1 ± 0.6h	1.9 ± 0.6fghij	0.14 ± 0.06e	9.0 ± 1.41jk	12.22 ± 0.3a	5.39 ± 0.12i	41.2
HN52	90	7.8 ± 1.6ghij	22.6 ± 1.8ab	14.8 ± 1.4defgh	8.1 ± 2.efgh	3.6 ± 1.5fghi	1.7 ± 0.4efgh	3.6 ± 1.0cdef	2.3 ± 0.8efgh	0.24 ± 0.08bcde	12.1 ± 1.1cd	10.23 ± 0.3bc	6.41 ± 0.12cdef	60.2
LC69	83.3	6.8 ± 1.6hij	17.1 ± 1.3gh	13.2 ± 0.8jk	4.6 ± 1.6j	2.6 ± 2.2hi	1.2 ± 0.7mpqrst	1.9 ± 0.7h	1.3 ± 0.6ij	0.14 ± 0.06e	9.4 ± 1.25ij	12.69 ± 0.3a	5.87 ± 0.12gh	59.5
HH76	95	6.3 ± 1.8j	19.6 ± 1.6def	15.0 ± 2.def	5.2 ± 1.8ij	2.9 ± 1.3ghi	1.4 ± 0.5gh	2.3 ± 0.8gh	1.6 ± 0.5ghij	0.15 ± 0.04e	11.6 ± 1.68def	11.93 ± 0.3a	6.11 ± 0.12efgh	54.7
LC26	75	5.8 ± 1.8nopqrst	18.9 ± 3.3ef	15.6 ± 2.3cde	4.4 ± 2.0j	2.5 ± 1.5i	1.2 ± 0.5h	1.9 ± 1.1h	1.0 ± 0.4j	0.17 ± 0.04de	10.3 ± 1.37gh	8.05 ± 0.5f	5.96 ± 0.20gh	35.3
Coefficient of variation	0.48	0.37	0.14	0.13	0.49	0.47	0.54	0.18	0.56	0.7	0.09	0.1	0.16	0.24

Different lowercase letters in the same column indicate significant differences at *p* < 0.05.

**Table A5:** Common factor variances of seed and fruit morphological indicators in principal component analysis (initial and extracted values).

Common Factor Variance	Initial	Extract
Seed length (mm)	1	0.665
Seed width (mm)	1	0.159
Seed thickness (mm)	1	0.204
Fruit length (cm)	1	0.863
Fruit width (mm)	1	0.656
Fruit thickness (mm)	1	0.492
Fruit weight (g)	1	0.97
Number of seeds (grain)	1	0.805
Fruit shell weight (g)	1	0.591
Heavy flesh (g)	1	0.859
Seed weight (g)	1	0.866
Silk weight (g)	1	0.752

Extraction method: Principal Component Analysis (PCA).

**Table A6:** ANOVA of selected physiological indicators of tamarind.

Cultivar	POD (u/g)	SOD (u/g)	Soluble Protein (mg/g)	Flavone (mg/g)	Titratable Acidity (mg/g)	Soluble Sugar (Pulp, mg/g)	Tamarind Polysaccharide (g/g)	Soluble Sugar (Leaves, mg/g)	Starch (Leaves, mg/g)
MDL2	50.53 ± 6.99k	1578.86 ± 32.49de	4.41 ± 0.37abcd	107.46 ± 5.25a	15.71 ± 0.94de	338.84 ± 48.65cd	0.46 ± 0.03ab	62.06 ± 3.28a	34.99 ± 1.91cd
HN52	957.4 ± 149.11de	1686.27 ± 34.82abcd	2.42 ± 0.49efgh	30.02 ± 11.84hij	13.54 ± 0.94efg	375 ± 6.14bc	0.45 ± 0.03abcd	29.24 ± 7fgh	42.37 ± 4.06bc
DH29	794.07 ± 120.61efg	1323.05 ± 244.44e	1.9 ± 0.43fghi	21.58 ± 1.62ij	17.5 ± 4.33de	393.72 ± 31.23abc	0.45 ± 0.01abc	7.32 ± 1.51k	12.58 ± 5.34g
TG94	349.87 ± 77.32ijk	1773.78 ± 13.6ab	4.53 ± 0.16abc	87.65 ± 3.73b	8.67 ± 0.94fg	377.21 ± 17.71bc	0.42 ± 0.03bcde	54.83 ± 1.85ab	28.58 ± 2.87def
DH14	141.33 ± 123.86jk	1784.06 ± 38.82ab	3.57 ± 0.13bcde	58 ± 6.06def	11.92 ± 0.94efg	351.52 ± 13.58	0.34 ± 0.01fg	43.81 ± 5.1cd	46.69 ± 5.09b
BS21	319.53 ± 246.22ijk	1728.9 ± 17.72abcd	5.33 ± 0.1a	71.38 ± 5.37bcd	11.92 ± 0.94efg	414.18 ± 12.68ab	0.37 ± 0.01ef	40.26 ± 3.16cde	28.7 ± 3.45def
HH76	38.6 ± 2.46k	1744.64 ± 29.68abc	4.09 ± 0.17abcd	68.99 ± 6.63cde	11.92 ± 0.94efg	412.9 ± 8.36ab	0.38 ± 0.05ef	47.36 ± 2.7bc	45.36 ± 1.46b
HN53	500.27 ± 107.82fghij	1338.47 ± 59.59e	1.21 ± 0.17hi	16.71 ± 7.4j	13.54 ± 0.94efg	369.77 ± 24.44bc	0.5 ± 0.05a	23.34 ± 5.81ghij	32.28 ± 3.45def
YJ80	1188.67 ± 86.74d	1633.42 ± 57.49bcde	1.68 ± 0.16ghi	26.71 ± 5.03ij	8.13 ± 1.63g	441.74 ± 10.53a	0.38 ± 0.05def	27.01 ± 0.73fghi	32.83 ± 0.56def
PZH71	379.13 ± 7.18hijk	1581.83 ± 23.18de	3.04 ± 0.42cdefg	16.41 ± 3.54j	13.54 ± 0.94efg	303.04 ± 14.31de	0.38 ± 0.01ef	18.94 ± 3.76ij	23.59 ± 0.87f
PE55	1964.67 ± 365.82b	1601.02 ± 22.3cde	1.49 ± 0.16hi	11.01 ± 0.82j	14.63 ± 1.63de	277.69 ± 38.85e	0.34 ± 0.04fg	16.53 ± 0.63j	33.13 ± 8.5def
QJYZ	1518.2 ± 308.61c	1739.13 ± 5.12abcd	3.51 ± 0.33bcde	82.86 ± 16.23bc	19.5 ± 1.63d	294.2 ± 11.53de	0.37 ± 0.02ef	53.57 ± 11.18ab	23.5 ± 0.6f
MDL1	559.6 ± 49.38fghi	1490.2 ± 220.74d	3.6 ± 1.02bcde	24.85 ± 15.52ij	16.25 ± 4.33de	396.63 ± 15.04abc	0.38 ± 0.06ef	20.69 ± 9.43hij	25.88 ± 2.26def
LC69	977 ± 263.55de	1732.31 ± 71.67abcd	0.88 ± 0.6i	19.71 ± 3.69ij	15.71 ± 2.48de	308.5 ± 10.62de	0.4 ± 0.03cdef	20.45 ± 1.36hij	24.75 ± 1.76ef
YM2	873.67 ± 371.04def	1772.07 ± 42.81ab	2.56 ± 0.99efgh	53.82 ± 16.86defg	15.17 ± 1.88de	351.98 ± 48.97cd	0.37 ± 0.04ef	36.1 ± 9.13def	11.04 ± 5.75g
LC26	472.07 ± 114.85ghij	1828.62 ± 11.87a	3.46 ± 0.76bcde	50.2 ± 1.44efg	14.08 ± 1.88def	367.68 ± 10.66bc	0.29 ± 0.02g	43.21 ± 2.26cd	79.59 ± 4.64a
TG110	749.13 ± 74.29efgh	1757.89 ± 83.16abc	4.75 ± 2.77ab	50.85 ± 23.76efg	14.08 ± 1.88def	344.19 ± 18.69cd	0.43 ± 0.03bcde	34.66 ± 9.66def	34.27 ± 5.78cde
YJ81	566.07 ± 206.7fghi	1744.64 ± 38.59abc	3.34 ± 0.55bcdef	46.82 ± 16.8fgh	17.33 ± 0.94a	369.19 ± 45.48bc	0.43 ± 0.03bcde	16.9 ± 6.42ij	24.14 ± 12.48f
YD12	555.87 ± 91.5fghi	1715.26 ± 41.34abcd	2.95 ± 0.18defg	53.59 ± 2.5defg	7.58 ± 1.88b	381.05 ± 85.98bc	0.37 ± 0ef	33.94 ± 2.98def	50.72 ± 3.81b
TG119	2389.4 ± 381.11a	1684.57 ± 5.91abcd	1.98 ± 0.16fghi	38.46 ± 5.24ghi	25 ± 4.33c	292.8 ± 20.54de	0.46 ± 0.04abc	31.59 ± 0.58def	32.25 ± 8.27def
Coefficient of Variation	0.81	0.09	0.46	0.59	0.28	0.14	0.14	0.46	0.46

Different lowercase letters in the same column indicate significant differences at *p* < 0.05.

**Table A7:** Common Factor Variances of Various Biochemical Indicators Under Principal Component Analysis (PCA, Initial & Extracted Values).

Common Factor Variance	Initial	Extract
Flavone (mg/g)	1	0.924
POD (u/g)	1	0.661
SOD (u/g)	1	0.669
Soluble protein (mg/g)	1	0.695
Titratable acidity (mg/g)	1	0.751
Soluble sugar (pulp, mg/g)	1	0.696
Tamarind polysaccharide (g/g)	1	0.7
Soluble sugar (leaves, mg/g)	1	0.857
Starch (leaves, mg/g)	1	0.559

Extraction method: Principal Component Analysis (PCA).
